# Exploration of a simplified clinical examination for scabies to support public health decision-making

**DOI:** 10.1371/journal.pntd.0006996

**Published:** 2018-12-27

**Authors:** Michael Marks, Daniel Engelman, Lucia Romani, Daniel Mason, Oliver Sokana, Mike Kama, Margot Whitfeld, Andrew C. Steer, John Kaldor

**Affiliations:** 1 Clinical Research Department, Faculty of Infectious & Tropical Diseases, London School of Hygiene and Tropical Medicine, London, United Kingdom; 2 Hospital for Tropical Diseases, London, United Kingdom; 3 Centre for International Child Health, University of Melbourne, Melbourne, Australia; 4 Tropical Diseases Research Group, Murdoch Children’s Research Institute, Melbourne, Australia; 5 The Kirby Institute, University of New South Wales, Sydney, Australia; 6 Solomon Islands Ministry of Health and Medical Services, Honiara, Solomon Islands; 7 Fiji Ministry of Health and Medical Services, Suva, Fiji; 8 St Vincent’s Hospital SydneyHospital, University of New South Wales, Sydney, Australia; Watford General Hospital, UNITED KINGDOM

## Abstract

**Introduction:**

In most settings, the diagnosis of scabies is reliant on time-consuming and potentially intrusive clinical examination of all accesible regions of skin. With the recent recognition of scabies as a neglected tropical disease by the World Health Organization there is a need for standardised approaches to disease mapping to define populations likely to benefit from intervention, and to measure the impact of interventions. Development and validation of simplified approaches to diagnose scabies would facilitate these efforts.

**Methods:**

We utilised data from three population-based surveys of scabies. We classified each individual as having scabies absent or present overall, based on whole body assessment, and in each of 9 regions of the body. We calculated the sensitivity of diagnosing the presence of scabies based on each individual body region compared to the reference standard based on whole body examination and identified combinations of regions which provided greater than 90% sensitivity. We assessed the sensitivity according to gender, age group, severity of scabies and the presence or absence of impetigo.

**Results:**

We included 1,373 individuals with scabies. The body regions with highest yield were the hands (sensitivity compared to whole body examination 51.2%), feet (49.7%), and lower legs (48.3%). Examination of the exposed components of both limbs provided a sensitivity of 93.2% (95% CI 91.2–94.4%). The sensitivity of this more limited examination was greater than 90% regardless of scabies severity or the presence or absence of secondary impetigo.

**Discussion:**

We found that examination limited to hands, feet and lower legs was close to 90% for detecting scabies compared to a full body examination. A simplified and less intrusive diagnostic process for scabies will allow expansion of mapping and improved decision-making about public health interventions. Further studies in other settings are needed to prospectively validate this simplified approach.

## Author summary

Scabies, caused by infestation with the microscopic mite *Sarcoptes scabiei*, is a major public health problem worldwide, particularly in low- and middle-income tropical settings. The diagnosis of scabies is reliant on detection of characteristic signs on clinical examination. Examination of the whole body is time-consuming and intrusive, whereas a more limited examination might be sufficient to guide public health decisions. We analysed data from several large scabies prevalence surveys to see if a more limited examination of the body provided acceptable sensitivity. We found that limiting examination to the exposed components of both limbs had high sensitivity compared to full body examination. Further studies are needed to prospectively validate simplified diagnostic approaches and aid scale up of scabies control programmes.

## Introduction

Scabies, a skin condition due to the microscopic mite *Sarcoptes scabiei* [1], is a major public health problem worldwide, particularly in low and middle income tropical settings, and has recently been adopted as a neglected tropical disease by the World Health Organization (WHO) [2]. This designation arose because of the increasing recognition of the importance of scabies, as well as the emerging evidence that effective control can be achieved by the strategy of mass drug administration (MDA). Evidence from studies using permethrin and ivermectin have demonstrated that MDA has a substantial impact on the prevalence of both scabies and secondary bacterial skin infections (impetigo) at the community level [3–6].

The scabies mite can only be directly visualised with a microscope, and there is currently no laboratory test for infestation. Therefore, the diagnosis of scabies is most often reliant on detection of characteristic signs on clinical examination, with only a limited role for direct visualisation in high resource settings. Scaling up MDA for scabies control will require a substantial effort in disease mapping to define populations and communities likely to benefit from intervention and then determining the impact of interventions. Development and validation of a simplified approach to diagnosis of scabies would facilitate these efforts.

In the clinical setting, the purpose of scabies diagnosis is to support optimal decision-making about individual patient management. As such, a thorough examination, that generally covers the entire skin surface, is required to minimise errors in diagnosis. However, for public health decisions, such as whether or not to initiate MDA, the aim is to assess the community prevalence of scabies, so a simplified examination might be appropriate, as has been used for other NTDs [7]. Such a protocol, if valid at the community level, could speed up data collection and reduce the imposition on survey participants that comes with full body examination which can be a barrier to accepting examination in some settings.

Therefore, we aimed to evaluate the accuracy of a simplified examination for the diagnosis of scabies that could be used to guide public health decision making.

## Methods

### Data sources

We utilised data from three recent, large population-based surveys of scabies, two conducted in the Solomon Islands, in Western and Choiseul provinces respectively, and one in Fiji.[6,8]. These studies were all population-based prevalence surveys using similar diagnostic and data collection tools. The surveys in Choiseul and Fiji were conducted as baseline for intervention trials. In all three studies examination involved examination of arms, legs, face and torso (excluding the breasts in women). Patients were asked if they had itch in the groin, buttocks or breasts and, if so, these areas were also examined. The whole body was examined in children aged under 1 year. Examination for all surveys was performed by individuals with experience in the diagnosis of scabies in low-resource settings.

**Fig 1:**
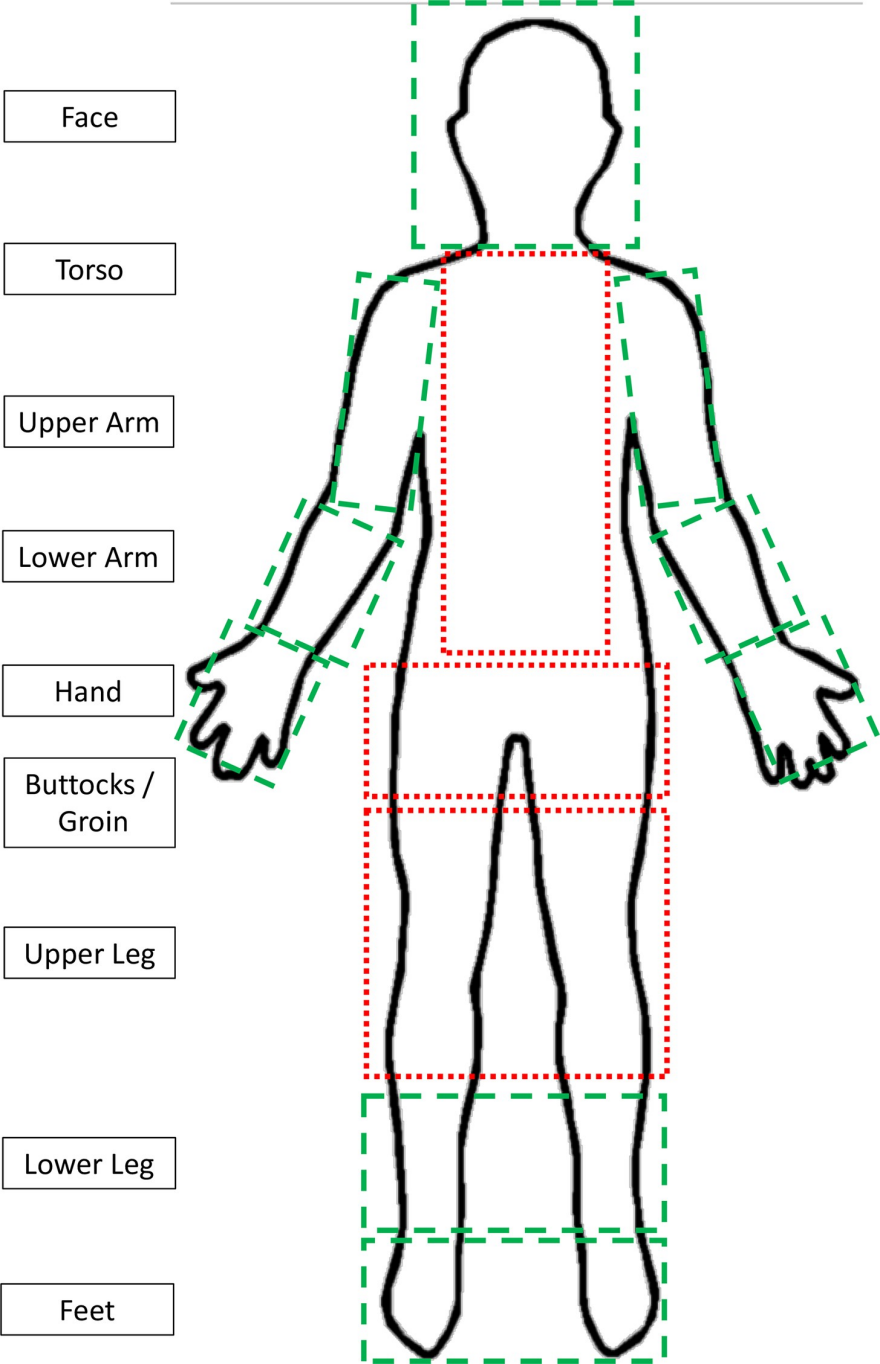
Classification of body regions. Clinical findings were classified into nine body regions. The upper arm included the axilla and the elbow. The lower arm included the wrist. The upper leg included the hip and knee. The lower leg included the ankle. For the purposes of this study areas that were classified as exposed are shown using a dashed-line and those that were classified as unexposed using a dotted-line.

From each survey’s primary database, we extracted patient-level data, including demographic characteristics, the presence or absence of both scabies and impetigo (bacterial infection) lesions and, if present, their number and distribution. Each study had used similar diagnostic criteria for scabies based on the finding of typical lesions (burrows, papules, nodules, vesicles) in a classical distribution[9]. In both the original studies and the combined analysis presented here, scabies severity was defined by the number of lesion detected as mild (≤10 lesions over all areas examined), moderate (11–49 lesions) or severe (≥ 50 lesions or crusted scabies)[9]. In each study, lesions which were moist, purulent or crusted were considered to indicate the presence of impetigo. In each of the original studies, examination findings were recorded for each of nine body regions **(Fig 1)**.

### Analysis

To obtain a reference diagnosis, we classified each individual as having scabies or not, according to whether scabies had been detected at any body site, using the standard examination conducted in the original studies. We further classified those with scabies, based on the presence or absence of scabies at each of the nine regions. We defined body regions as “exposed” if they could be routinely examined without removing clothes. The face, the upper arm (including the elbow) and lower arm (including the wrist), hands and the lower leg (including the ankles) and feet met this definition. The torso, upper legs (including the knee), buttocks and groin were the “unexposed” regions.

We then evaluated simplified diagnostic algorithms based on body region-specific findings: A person would be classified as having scabies if it was detected at a particular region or grouping of regions. For these algorithms, we calculated the sensitivity compared to the reference standard based on whole-body examination. We then identified groupings which provided greater than 90% sensitivity in comparison to the reference standard. We assessed sensitivity across subgroups defined by gender, age group, severity of scabies and the presence or absence of impetigo. We calculated the prevalence of scabies that would have been measured in each of the three original studies using optimal combinations based on simplified examination. We used a one-sided test to compare the proportion of individuals diagnosed with scabies based on the standard examination with the proportion diagnosed based on an examination of ‘exposed’ body regions. We considered a p-value of <0.05 to be consistent with a statistically significant difference. Statistical analysis was performed in R 3.4.3 (The R Foundation for Statistical Computing).

## Results

The combined sample size of the three study datasets was 5,358, with similar numbers contributed from each of the three surveys (1908, 1399 and 2051 from Western and Choiseul provinces of the Solomon Islands, and Fiji). Overall 2,801 (52.3%) of study participants were female and the median age was 14 years (IQR 7–36 years) **(Table 1).**

**Table 1:** Demographic characteristics of participants in three surveys of scabies prevalence.

		n	%
**Gender**	Male	2557	47.7%
	Female:	2801	52.3%
**Age**	0-4yrs:	763	14.2%
	5-9yrs:	1026	19.1%
	10-14yrs:	904	16.9%
	15-24yrs:	491	9.2%
	25/49yrs:	1412	26.4%
	50+yrs:	692	12.9%
**Scabies**	Absent	3985	74.4%
	Present	1373	25.6%
**Scabies Severity**	N/A	3985	74.4%
	Mild	684	12.8%*
	Moderate	513	9.6%*
	Severe	176	3.2%*
**Impetigo**	Absent	3933	73.4%
	Present	1425	26.6%

*% of all individuals

In the original studies 1,373 individuals (25.6%) were diagnosed with scabies (18.1%, 18.7% and 36.4% across the surveys) at any body location. Scabies was present in a median of 2 body regions (IQR 1–3). Of the 1373 cases of scabies, the disease was classified as mild in 684 (49.7%) participants, moderate in 513 (37.5%) and severe in 176 (12.8%). Data on scabies severity was missing for two participants, so they were excluded from subgroup analyses. Overall the proportion of individuals with impetigo in the original studies was 26.6% (n = 1,425) and was significantly higher among individuals with scabies (45.1% vs 20.2%, OR 3.24, p <0.001).

The highest diagnostic yield was through examination of the hands (sensitivity compared to whole body examination 51.2%), feet (49.7%), and lower legs (48.3%). As shown in Table 2, examination of the whole of the upper limb (upper arm, lower arm and hand) had a sensitivity of 67.4% (95% CI 64.8–69.8%) compared to the reference standard examination. Examination of the exposed part of the lower limbs (lower leg and feet) had a sensitivity of 55.8% (95% CI 53.2–68.5%) compared to the reference standard examination. Examination of the exposed components of both limbs had sensitivity of 93.2% (95% CI 91.2–94.4%).

**Table 2:** Proportion of scabies cases identified by body region.

Body Site	n	Percentage of scabies cases with site involved	Exposed or Unexposed
Any Body Region	1373	100%	N/A
Face	46	3.4%	Exposed
Upper Arm	238	17.3%	Exposed
Lower Arm	400	29.1%	Exposed
Hand	703	51.2%	Exposed
Torso	194	14.1%	Unexposed
Buttock/Groin	94	6.8%	Unexposed
Upper Leg	310	22.6%	Unexposed
Lower Leg	663	48.3%	Exposed
Feet	682	49.7%	Exposed

The sensitivity of the algorithm based on exposed regions was above 90% across all subgroups defined by sex and age group except people over 50 years, in whom it was 88.0% (95% CI 81.3–92.7). It was greater than 90% in mild, moderate and severe scabies and individuals with or without impetigo (Table 3). Excluding the upper arms from the examination significantly reduced the sensitivity in a number of subgroups while examining the remaining exposed site, the face, did not significantly increase sensitivity **(Table 3)**.

**Table 3:** Sensitivity of limited examination across population subgroups.

		Lower Arms, Lower Legs	Whole Arms, Lower Legs	All exposed (Whole Arms, Lower Legs, Face)
**Gender**	Male	87.8 (85.0–90.1)*	92.9 (90.7–94.7)	93.6 (91.5–95.3)
	Female:	86.5 (83.4–88.9)*	92.0 (89.7–93.9)	94.4 (92.2–95.6)
**Age**	0-4yrs:	91.0 (86.7–94.1)	92.2 (88.0–95.0)	93.3 (89.4–95.9)
	5-9yrs:	91.7 (88.3–94.2)	94.5 (91.5–96.5)	95.3 (92.4–97.2
	10-14yrs:	94.5 (91.1–96.7)	95.9 (92.7–97.8)	96.6 (93.4–98.2)
	15-24yrs:	89.8 (81.0–94.9)	96.6 (89.7–99.1)	96.6 (89.7–99.1)
	25/49yrs:	80.6 (74.5–85.5)*	90.3 (85.3–93.7)	91.7 (86.9–94.8)
	50+yrs:	67.6 (59.2–75.0)*	88.0 (81.3–92.7)	88.7 (82.1–93.2)
**Impetigo**	Absent	84.5 (81.6–86.9)*	93.0 (90.8–94.6)	94.1 (92.2–95.7)
	Present	91.8 (89.3–93.8)	93.4 (91.1–95.2)	93.9 (91.6–95.6)
**Scabies Severity**	Mild	84.9 (82.0–87.5)*	91.4 (89.0–93.3)	92.7 (90.4–94.5)
	Moderate	88.1 (84.9–90.7)*	93.8 (91.2–95.6)	94.3 (91.9–96.1)
	Severe	97.8 (93.9–99.3)	98.3 (94.7–99.6)	98.3 (94.7–99.6)

*p<0.01 for comparison to sensitivity when including upper arm

The prevalence estimates derived from simplified examination did not differ significantly from those obtained in any of the original surveys. Excluding the upper arm resulted in a statistically significant difference in the prevalence estimate in a single survey **(Table 4)**.

**Table 4:** Prevalence estimates by simplified examination.

	n	Reference Standard Examination	Lower Arms, Lower Legs	Whole Arms, Lower Legs	Whole Arms, Lower Legs, Face
**Western Province, Solomon Islands**	1908	19.2	17.2	18.0	18.1
**Choiseul Province, Solomon Islands**	1399	18.7	16.4	17.4	17.4
**SHIFT Study,** **Fiji**	2051	36.4	31.5	33.7	34.2

## Discussion

Based on analysis of primary data from three large, population-based surveys of scabies prevalence, we found that restriction to particular body regions defined as exposed had close to 90% sensitivity for detecting scabies, compared to a whole-body examination. Use of a restricted examination would have generated prevalence estimates very similar to those obtained from full body examination. Importantly, this finding was not dependent on severity of scabies or the presence or absence of impetigo.

With scabies newly recognised as a neglected tropical disease by WHO, efforts are underway to identify and implement intervention strategies, potentially including MDA. In order to scale up interventions, it will be necessary to have standardised means of classifying geographic areas in regard to scabies prevalence. Best practice methods of assessment reported from recent prevalence surveys and trials have generally depended on whole body examination by experts in dermatology. This method has a number of limitations, including the need for private examination rooms, the time required, and participant sensitivities about examination. Defining and validating a simplified form of examination will facilitate mapping, especially in resource-limited settings. The approach of seeking simplifications in diagnostic processes has been used in the context of other NTDs such as the WHO grading criteria for trachoma [7], and allowed the large-scale mapping of disease prevalence [10].

Even more limited examination, such as the hands alone, had a sensitivity of only 51.2%. It might be argued that for public health decision-making, it is more important to provide a broad ranking of prevalence than to accurately estimate the absolute level, but the markedly reduced sensitivity of examining the hands alone would substantially increase the likelihood of making the wrong decision. More feasibly, an examination of both arms and both lower legs had greater than 90% sensitivity, providing an option that balances accuracy for public health purposes, while being practical in the field.

In all three studies that were the source of data for the analyses presented here, the diagnosis of scabies was made by an individual experienced in the diagnosis of scabies. We used current best available diagnostic criteria which have previously been validated in both the Pacific and Africa [9,11]. Potentially, the sensitivity of a more limited examination might be reduced if conducted by a person with less training or experience. It will be important to conduct further validation of simplified examination performed by those with less experience. Validation of the simplified criteria could be conducted alongside prospective validation of recently published consensus diagnostic criteria for scabies [12]. A crucial step in preparing for such validations will be the development of standardised training materials so that a much larger number of assessors can be engaged in evaluations of scabies prevalence, as has been done for trachoma grading [10].

A limitation of the data sources analysed is that the breasts and groin were only examined in the underlying surveys if participants reported itch. It is therefore possible that some people had scabies in these regions but were classified as not having scabies. The consequence of this would be an over-estimate of the sensitivity of a more limited examination. However, these differences would be unlikely to alter the prevalence estimates sufficiently to be of public health importance.

Our data are derived entirely from studies conducted in the Pacific region. Evaluation of the proposed simplified diagnostic approach will need to be conducted in a wider range of demographic and geographic settings to ensure the findings are broadly applicable. The extent to which areas of the skin are exposed and may be examined is to a large extent culturally dependent, and will therefore vary by region. For example, in some regions lifting up a sleeve to examine the lower portion of the upper arm may therefore be considered unobtrusive but lifting up a shirt to expose the abdomen less so. Further studies are necessary to evaluate our proposed simplified algorithm in a variety of epidemiologic settings, using prospective methodology. Further validation of the simplified assessment will need to consider both the accuracy, and acceptability of different levels of examination. Other issues, such as the gender of the examiner and setting of the examination, will also be relevant in ensuring that culturally appropriate methods of prevalence assessment are widely available in scabies-endemic areas.

The adoption of scabies as a neglected tropical disease by the WHO has provided fresh impetus to the development of tools to control scabies as a public health problem. Our study adds valuable data to the development of a simplified diagnostic process for scabies that may be applied to guide decisions about future public health interventions.
